# Brain Monoamine Deficits in the CD Mouse Model of Williams–Beuren Syndrome

**DOI:** 10.3390/biom15101382

**Published:** 2025-09-28

**Authors:** Chloé Aman, Hélène Gréa, Alicia Rousseau, Anne-Emilie Allain, Susanna Pietropaolo, Philippe De Deurwaerdère, Valérie Lemaire

**Affiliations:** Institut de Neurosciences Cognitives et Intégratives (INCIA, UMR5287), Centre National de la Recherche Scientifique, Université de Bordeaux, F-33000 Bordeaux, France; chloe.aman@u-bordeaux.fr (C.A.); helene.grea@u-bordeaux.fr (H.G.); alicia.rousseau.alicia@gmail.com (A.R.); anne-emilie.allain@u-bordeaux.fr (A.-E.A.); susanna.pietropaolo@u-bordeaux.fr (S.P.); valerie.mayo@u-bordeaux.fr (V.L.)

**Keywords:** dopamine, serotonin, noradrenaline, connectivity, correlation, metabolism, neurochemistry, nucleus accumbens, cortex, hypothalamus

## Abstract

Williams–Beuren Syndrome (WBS) is a rare neurodevelopmental disease caused by a microdeletion on chromosome 7 (7q11.23) and associated with behavioral disorders such as hypersociability, impaired visuospatial memory, anxiety, and motor disorders. The precise underlying neurobiological bases remain unknown. The CD mouse is a genetic model that reproduces the deletion found in WBS patients on the equivalent mouse locus. Taking into account that monoaminergic systems are known to modulate behaviors that are altered in WBS, we hypothesized that CD mice could present quantitative and qualitative changes in brain noradrenaline, dopamine, and serotonin systems compared to wild-type (WT) littermates. We sampled 10 brain regions in female mice for quantifying monoamines and related compounds by high-performance liquid chromatography coupled to electrochemical detection. We found a decrease in dopamine in the nucleus accumbens and serotonin and its metabolites in the hypothalamus. Using correlative approaches of tissue content across the brain, we found that the relationships between neurotransmitters or their metabolic ratios (metabolite/neurotransmitter) changed in CD compared to WT. Notably, compared to WT, the ratios in CD mice showed striatal correlations for the serotonin/dopamine systems interaction, and cortical, thalamic, and hypothalamic correlations for the noradrenaline/dopamine systems interaction. The data suggest specific alterations of monoaminergic systems across the brain that could sustain the abnormal behavioral responses displayed by CD mice.

## 1. Introduction

Williams–Beuren syndrome (WBS) is a rare genetic disorder characterized by intellectual disability, heart defects, and distinctive physical and behavioral characteristics [1,2]. The behavioral profile of WBS individuals is characterized in particular by hypersociability, cognitive impairment of varying severity, and impaired visuospatial abilities. It also includes some motor disturbances that have been related to extrapyramidal signs [3]. The genetic mutation associated with this disorder is a microdeletion on chromosome 7, which contains several genes essential for neurosensory and cardiovascular functions [4]. Rare partial forms of WBS are linked to smaller deletions and patients with reciprocal microduplication of the same 7q11.23 region have also been identified [5]. The genetic deletion results in the monoallelic loss of 28 genes, some of which have been identified including the transcription factor *gtf2ird1* [4,5], mostly involved in the neurobehavioral phenotype of WBS. The widespread nature of the behavioral alterations suggests that several neurobiological networks are altered [2,6], but this is still not well known.

A genetic mouse model mimics the complete heterozygous deletion found in humans with WBS. This “complete deletion” (CD) mouse model reproduces certain phenotypes frequently observed in WBS patients [7]. The CD mouse model, beyond cardiovascular abnormalities [7,8,9], displayed early and adult behavioral alterations including altered social interaction and communication, delayed sensory development, alteration of exploratory and anxiety-like behaviors, and sensorial sensitivity [7,10] as well as cognitive deficits in adulthood [11,12,13,14,15]. Like the human disease [16], these deficits were equally found in both CD male and female mice [14] with few sex-dependent effects, mostly observed in young mice only [10]. Thus, the model could permit to determine neurobiological alterations over several brain functional networks.

The monoamines noradrenaline (NA), dopamine (DA), and serotonin (5-HT) act as neuromodulators in the central nervous system (CNS) and participate in the organization and function of neurobiological networks during development and in adulthood. All these monoaminergic neurons diffusely project throughout the CNS from clusters of cell bodies located in the brainstem and regulate several behavioral functions [17,18,19,20,21]. Their dysregulation has been postulated in WBS patients, particularly for the DA system [3], but poorly studied either in humans or in animal models [11,22]. Tissue monoamine contents at rest allows for measuring quantities of monoamines and associated compounds (metabolites/precursors) in several brain structures, and approximating connectivity of monoaminergic function across sampled brain regions [23,24]. We therefore hypothesized that subtle dysregulations of monoamine contents and connectivity across several brain regions may be present in CD mice, possibly underlying their behavioral deficits.

In the present study, we assessed the post mortem brain distribution of NA, DA, and 5-HT and associated compounds in CD and WT adult mice. We selected 10 brain regions based on their involvement in the behavioral disorders found in this model (see Section 2.2). Tissue levels of monoamine as well as their metabolites and precursors were measured using high performance liquid chromatography coupled to electrochemical detection (HPLC–EC) technique.

## 2. Materials and Methods

### 2.1. Animals

A cohort of 7 CD mice and 9 WT female littermates, aged 7 months and behaviorally naive, was used for this study. They were obtained by crossing heterozygous CD male mice bred in the animal facility of the University of Bordeaux with WT C57BL/6J female mice purchased from Janvier (Le Genest-Saint-Isle, France; authorization # 34602-2022011017347236 to S.P. from the Ethical Committee for Animal Experimentation of the University of Bordeaux CEEA 050; 4 March 2022). The CD colony was derived from CD breeders kindly provided to S.P. by Dr. Victoria Campuzano from Barcelona University. The choice of the female sex and the number of animals were conditioned by limitations in mouse availability. Despite these limitations, the well-known lack of major sex differences in the behavioral phenotype of adult CD mice [14] was taken as one criterion to conduct this neurochemical experiment in females only. The experiments were performed in animals from a single cohort, as recommended by recent guidelines on brain monoamine quantification [23]. Each mouse was marked at one week of age by a paw tattoo with a non-toxic and odorless permanent ink and genotyped as previously described [10]. Mice were group-housed since weaning under standard conditions (T°: 21 ± 1 °C, 12-h light/dark cycle, housed in polycarbonate cage enriched with nesting material) with access to water and food ad libitum.

### 2.2. Selection of Brain Regions

Mice were sacrificed one by one in an isolated room by cervical dislocation during the light phase. Their brains were immediately extracted and frozen in isopentane cooled by dry ice for 3 min. The brains were then stored in tubes at −80 °C. To sample the structures of interest, the brains were cut in the coronal plane in a cryostat at −22 °C. The following 10 regions were selected for our study: the prelimbic cortex (PL) for its role in planning and working memory; the orbitofrontal cortex (OF) for its involvement in social behaviors; the amygdala (A) for its role in anxiety; the auditory cortex (AuC) for its role in sound processing; the thalamus (T) for its sensory integration function; the ventral (vH) and dorsal (dH) hippocampus for their involvement in spatial memory encoding and retrieval; the nucleus accumbens (NAc) and striatum (STR) for their roles in motor planning and motivation; and the hypothalamus (Hy) for its key role in the interface between higher nervous functions and the periphery via the control of the autonomic system and social interactions. The different regions of interest were sampled as previously reported [25] with some modifications. The bilateral punches were made blind of the genotype using 600 µm stainless steel cannula for OF, PL, AuC, A, dH, vH, T, Hy, and 800 µm stainless steel cannula for NAc and STR. The samples were placed in pre-weighed 0.5 mL Eppendorf tubes. The tubes were then stored at −80 °C until the biochemical analysis.

### 2.3. Neurochemical Analysis

The HPLC–EC apparatus was composed of an HPLC column (Hypersyl, 15 cm × 4.6 mm, C.I.L. Sainte Foy la Grande, France) and a mobile phase as previously reported [26]. The mobile phase is composed of methanol (7%), triethylamine (100 µL/L), NaH_2_PO_4_ (70 mM), EDTA (0.1 mM), sodium octyl sulfate (100 mg/L), and deionized water (18.2 MΩ.cm), and the pH was adjusted (approximately 4.2) with phosphoric acid to achieve good separation between compounds. The mobile phase was filtered (0.22 µm) and was delivered at a flow rate of 1.3 mL/min using a pump (LC20AD, Shimadzu, Paris, France). Sample injection was performed using a manual injection valve (Rheodyne, model 7725i, C.I.L. Sainte Foy la Grande, France) equipped with a 20 µL loop. Compounds of interest were eluted at different retention times to the electrochemical detection cell equipped with oxidation and reduction electrodes set at 350 mV and −270 mV, respectively. The coulometric detector (Coulochem II, ESA, Paris, France) was connected to a computer via an interface (Ulyss, Azur 5.0, Datalys, Saint-Martin-d’Hères, France). Under these conditions it is possible to measure NA, DA, and 5-HT as well as their metabolites, vanylmandelic acid (VMA), dihydroxyphenylacetic acid (DOPAC), homovanillic acid (HVA), 5-hydroxyindoleacetic acid (5-HIAA), and the precursors 3,4-dihydroxyphenylalanine (L-DOPA) and 5-hydroxytryptophan (5-HTP). The emphasis was given to the monoamines NA, DA, and 5-HT and the ratios DOPAC/DA, 5-HIAA/5-HT, and VMA/NA which correspond to indirect indexes of the neurotransmitter turnover rate. The chromatographic conditions were checked by calibrations with standards solution made at different concentrations depending on the regions studied.

On the day of the neurochemical analysis, the tubes containing the brain region of interest were removed from −80 °C and placed on ice. Each tube was wiped off its water and then reweighed on the same precision balance as for the pre-weighing to determine the mass of collected tissue. An amount of 100 µL of perchloric acid (HClO4, 0.1 N, 4 °C) was added to the tube and the tissue was homogenized by sonication and centrifuged for 30 min at 13,000 rpm at 4 °C. 10 µL of the supernatant was injected in the HPLC–EC system. Samples were injected and analyzed blind to the animal genotype. The final amount of compound was normalized to the tissue weight expressed in mg.

### 2.4. Statistical Analysis

Data from the WT and CD groups were analyzed using the Grubbs test to eliminate possible outliers (only one outlier permitted). A Shapiro–Wilk normality test was performed to verify that the majority of the data followed a normal distribution in considering each compound and the different genotype. A Student’s *t*-test was thus performed to compare the means of the two groups.

For correlations, the coefficient r of Pearson was used to study the correlations of the neurotransmitter content (or ratio) between brain regions for each genotype, alone or in combination with another neurotransmitter (or another ratio).

In all cases, the significance criterion was set at *p* value < 0.05. All statistical analyses were performed using GraphPad 9.0 software.

## 3. Results

### 3.1. Quantitative Profile of Monoaminergic Systems Between WT and CD Mice in Different Brain Regions

**Figure 1:**
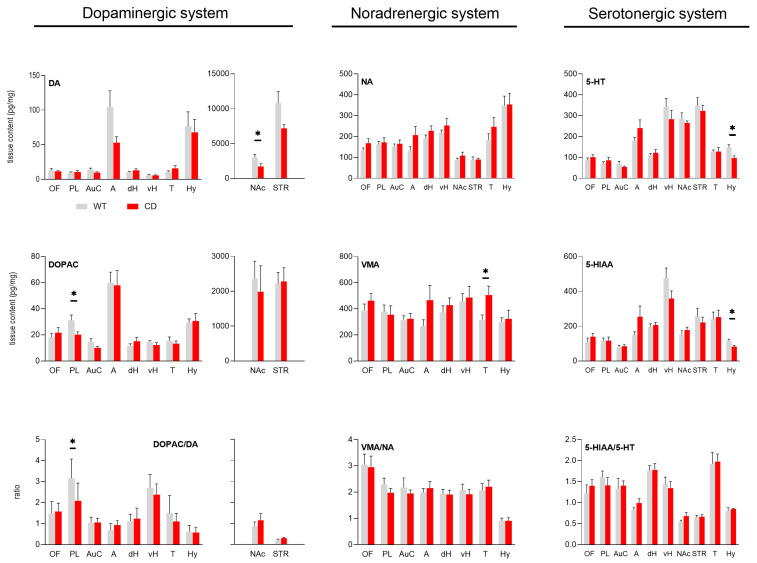
Tissue content of monoamines (upper panels), respective metabolites (middle panels), and ratios (lower panels) in WT and CD mice. Bars of histogram (grey for WT; red for CD mice) represent the values (expressed in pg/mg, mean ± SEM) of monoamines (DA, NA, 5-HT) and their metabolites (DOPAC, VMA, 5-HIAA), and ratios (DOPAC/DA, VMA/NA, 5-HIAA/5-HT) for the ten explored brain regions (OF: orbitofrontal cortex, PL: prelimbic cortex, AuC: auditory cortex, A: amygdala, dH: dorsal hippocampus, vH: ventral hippocampus, NAc: nucleus accumbens, STR: striatum, T: thalamus, Hy: hypothalamus). For the dopaminergic system (left panels), all values in NAc and STR are represented on a separate graph due to the high concentration of DA and DOPAC in these structures (for the sake of homogeneity, DOPAC/DA ratios in NAc and STR are also represented apart). Tissue contents were compared using Student’s *t*-test. * *p*-value < 0.05, *n* = 7 (CD) and 9 (WT) animals/group. VMA was not measured in NAc and STR.

Quantitative analysis of the tissue levels of monoamines is reported in Figure 1 for the brain region analyzed.

#### 3.1.1. Dopaminergic System

The tissue levels of DA were similar for both genotypes in several brain regions. They were lower in the amygdala, nucleus accumbens and striatum of CD mice when compared to WT mice, but the difference was significant in nucleus accumbens only (Figure 1, upper left panel, DA: WT = 3042 ± 412 pg/mg, CD = 1718 ± 396 pg/mg, *p* = 0.04). However, no statistical difference was reported for its two metabolites (DOPAC: WT = 2360 ± 502 pg/mg, CD = 1985 ± 745 pg/mg, NS; HVA: WT = 625 ± 73 pg/mg, CD = 589 ± 115 pg/mg, NS; Supplementary File, Figure S1). A significant decrease in DOPAC was observed in prelimbic cortex in CD mice when compared to WT mice (Figure 1, middle left panel, DOPAC: WT = 31 ± 4 pg/mg, CD = 20 ± 2 pg/mg, *p* = 0.044). Moreover, HVA tissue content was significantly lower in the ventral hippocampus of CD mice (Supplementary File Figure S1, left panel, HVA: WT = 29 ± 3 pg/mg, CD = 20 ± 2 pg/mg, *p* = 0.042). The DOPAC/DA ratio in nucleus accumbens tended to be higher in CD mice without reaching statistical significance (DOPAC/DA: WT = 0.88 ± 0.2, CD = 1.15 ± 0.3, NS). In prelimbic cortex, however, DOPAC/DA ratio was significantly lower in CD mice when compared to WT mice (Figure 1, lower left panel, DOPAC/DA: WT = 3.16 ± 0.3, CD = 2.09 ± 0.3, *p* = 0.031). Finally, the levels of L-DOPA were similar in WT and CD mice in all explored brain regions (Supplementary File Figure S1, middle panel).

#### 3.1.2. Noradrenergic System

NA tissue contents were similar in all brain regions explored between WT and CD mice (Figure 1, upper middle panel). However, the tissue level of the NA metabolite VMA was higher in the thalamus in CD mice (Figure 1, middle panel, VMA: WT = 316 ± 37 pg/mg, CD = 506 ± 68 pg/mg, *p* = 0.024). Finally, the ratio VMA/NA was similar between CD and WT mice for all explored brain regions (Figure 1, lower middle panel).

#### 3.1.3. Serotonergic System

The 5-HT tissue contents were similar between WT and CD mice in all explored brain regions except in the hypothalamus where they were lower in CD mice when compared to WT mice (Figure 1, right upper panel, 5-HT: WT = 148 ± 13 pg/mg, CD = 97 ± 10 pg/mg, *p* = 0.02). In the hypothalamus, both precursor 5-HTP and metabolite 5-HIAA were decreased in CD mice (for 5-HTP see Supplementary File Figure S1, right panel, 5-HTP: WT = 7.9 ± 0.97 pg/mg, CD = 4.3 ± 0.52 pg/mg, *p* = 0.0118; for 5-HIAA see Figure 1. Right middle panel, WT = 116 ± 10 pg/mg, CD = 83 ± 8 pg/mg, *p* = 0.034). Finally, the ratio 5-HIAA/5-HT was similar between WT and CD mice in all considered brain regions (Figure 1, lower right panel).

### 3.2. Correlative Profile of Monoaminergic Systems Between the Different Brain Regions for WT and CD Mice

We first determined whether related compounds (neurotransmitter versus metabolite, or two connected metabolites) showed some correlations in each single brain region to determine the consistency of the dataset. The proportion of brain regions with significant correlations between metabolically related compounds reached between 40 and 70% (Supplementary File, Figure S2). The pattern of these relationships was slightly different in WT and CD mice. This proportion for VMA/NA, not previously described, slightly increased in CD mice.

**Figure 2:**
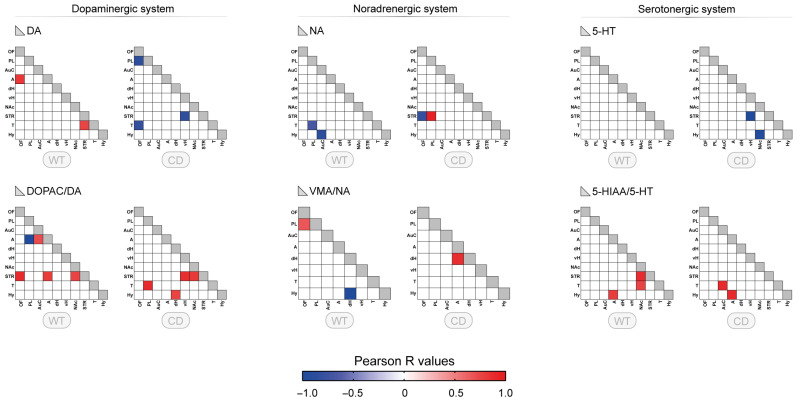
Correlative profile of monoaminergic systems between different brain regions for WT and CD mice. Correlative analysis of tissue content and turnover index (ratio) for the dopaminergic system (DA, DOPAC/DA), noradrenergic system (NA, VMA/NA), and serotonergic system (5-HT, 5-HIAA/5-HT) among the 10 brain regions studied for WT and CD mice (OF: orbitofrontal cortex, PL: prelimbic cortex, AuC: auditory cortex, A: amygdala, dH: dorsal hippocampus, vH: ventral hippocampus, NAc: nucleus accumbens, STR: striatum, T: thalamus, Hy: hypothalamus). Each colored square corresponds to a significant correlation (*p* < 0.05, r Pearson’s coefficient) with the color indicating the direction of the correlation (blue: negative, red: positive). The color gradient corresponds to the strength of the correlation (R values). VMA was not measured in STR and NAc, reducing to eight the number of regions for the ratio VMA/NA.

To fully estimate the neurochemical changes associated with CD genotype, correlative analyses were performed for each monoamine and their turnover among the 10 brain regions shown previously. For monoamines, only a few correlations were found (around two correlations), with no correlation for 5-HT in the WT group (Figure 2, upper panels). Changes in the number of correlations were observed for CD genotype, mostly for 5-HT. No clear regional pattern was noticed for either group. In contrast, more correlations were present for the turnovers with a higher number of correlations found for DOPAC/DA ratio in WT, involving both amygdala and striatum (A and STR: 3 correlations) (Figure 2, lower panels). In CD mice, the neurochemical pattern was altered for all turnovers. A specific alteration of the regional pattern was found for DOPAC/DA ratio with a suppression of the amygdala correlations towards other correlations including thalamus and hypothalamus.

### 3.3. Correlative Profile of Monoaminergic Systems Interaction Between the Different Brain Regions for WT and CD Mice

**Figure 3:**
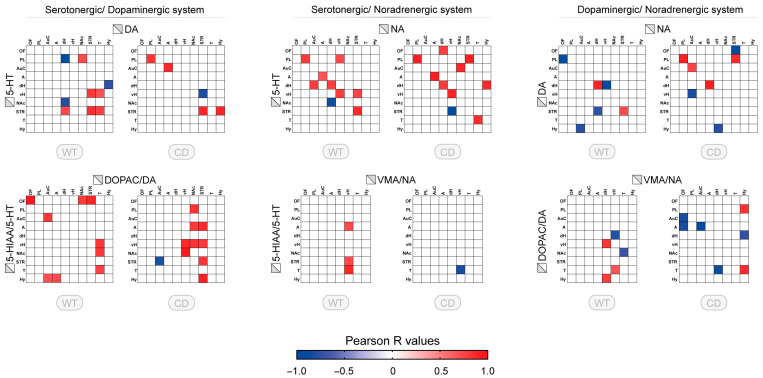
Correlative profile of monoaminergic systems between different brain regions and monoamine systems for WT and CD mice. Correlation matrix of tissue content and turnover index (ratio) between dopaminergic and serotonergic system (5-HT vs. DA, 5-HIAA/5-HT vs. DOPAC/DA), noradrenergic and serotonergic system (5-HT vs. NA, 5-HIAA/5-HT vs. VMA/NA), noradrenergic and dopaminergic system (DA vs. NA, DOPAC/DA vs. VMA/NA) in the eight or ten brain regions studied for WT and CD mice (OF: orbitofrontal cortex, PL: prelimbic cortex, AuC: auditory cortex, A: amygdala, dH: dorsal hippocampus, vH: ventral hippocampus, NAc: nucleus accumbens, STR: striatum, T: thalamus, Hy: hypothalamus). Each colored square corresponds to a significative correlation (*p* < 0.05, r Pearson’s coefficient) with the color indicating the direction of the correlation (blue: negative; red: positive). The color gradient corresponds to the strength of the correlation (R values).

Furthermore, we explored the interaction of the monoaminergic systems through correlation matrix of different combinations of monoaminergic systems across sampled brain regions. For the combination of monoamines, only a few correlations were found, with a higher proportion between DA and 5-HT or NA and 5-HT (nine correlations in WT) (Figure 3, upper panels). Reduced number of correlations and altered profile of correlations were reported for the correlations between 5-HT and DA in CD mice. Compared to WT mice, no correlation involving the nucleus accumbens, thalamus, or dorsal hippocampus was observed while those correlations involving the striatum were still present but one of them with a negative direction (STR/vH). The correlations between 5-HT and NA were quite maintained in the same brain regions (diagonal), but the other correlations changed. The correlations between NA and DA were approximately similar in number but only one in dorsal hippocamps was observed in both genotypes (Figure 3, upper panels, right side).

The analysis of the ratios across the brain suggests the presence of distinct alterations of the regional pattern in WT and CD genotypes (Figure 3, lower panels). For the interaction between 5-HT and DA ratios in WT, the correlations, all positive, mostly involved the orbitofrontal cortex, thalamus, and hypothalamus. In CD mice, the correlations between DA and 5-HT mostly involved the striatum and the nucleus accumbens. For 5-HT and NA ratios interaction, the correlations were limited to NA in ventral hippocampus with subcortical 5-HT. The sole correlation in CD mice (in vH/T) was inverted with respect to WT. The correlations between DOPAC/DA and VMA/NA were few, mostly in the ventral hippocampus and the dorsal hippocampus in subcortical regions. The pattern was more widespread in CD mice including cortical regions, amygdala, and hypothalamus, with a higher proportion of negative correlations (Figure 3, lower panels, right side).

## 4. Discussion

The results show specific alterations in monoamine quantities, subtle and restricted to a few brain regions. These slight alterations are also associated with a reorganization of the connectivity of monoaminergic systems. The results support the hypothesis that monoaminergic systems are modified both quantitatively and qualitatively in the CD mouse model of WBS.

The quantities of monoamines, metabolites, and precursors measured in the different brain areas were heterogeneous, mostly for the DA system, and were in the expected range of their concentration [25,26,27]. In quantitative terms, both the DA and the 5-HT systems displayed specific deficits in CD mice with respect to the NA system. For the latter, only the metabolite VMA was markedly increased in the thalamus of CD mice. The lower values for DA in the nucleus accumbens might not be related to a lower number of DA neurons in the ventral tegmental area because it was not associated with the modification of levels of DOPAC, HVA, and the DOPAC/DA ratio. This reduced DA tone may underlie the deficits in motor functions observed in adult CD mice of both sexes in the rotarod test [7,14].

The deficits in 5-HT, as well as its precursor 5-HTP and its metabolite 5-HIAA were restricted to the hypothalamus. The decrease in 5-HT specifically in the hypothalamus is putatively not associated with reduction of 5-HT neurons in raphe nuclei either. The anatomical data suggest some kind of specificity of the distribution of 5-HT neurons and their projections [28,29,30], but the neurons that innervate the hypothalamus are also innervating other brain regions we sampled. Moreover, it has been reported that the autoradiographic labelling of the 5-HT transporter using [125I]RTI-55 was not modified in CD mice, including the hypothalamus [11]. Our data are different from a previous study in mice lacking only one WBS gene, i.e., coding for the transcription factor *gtf2ird1* [31], describing a slight increase in the levels of 5-HIAA, but not 5-HT, in some cortical regions and the amygdala [22]. However, the comparison with the CD mouse line is difficult because the monogenic *gtf2ird1* model does not reproduce several features of WBS and is based on a different genetic background. Thus, the decrease of 5-HT levels in CD mice could correspond to lower activity of the whole metabolic pathway of 5-HT terminals in the hypothalamus, perhaps related to local changes with neurochemical partners.

The specific alterations in tissue levels of monoamines in CD mice are unique and could impact the whole neuromodulatory spectrum of monoamines in the brain. The only way to address this possibility to date is to study the connectivity of monoaminergic neurochemical markers across the brain via correlative analyses [23,24]. The data show a distinct profile of correlations between WT and CD mice across the brain notably as regards the dopaminergic system, alone or in combination with the serotonergic or noradrenergic system. As the data were obtained from mice at rest, it seems that the correlations of the ratios offer more informative value than the neurotransmitters themselves or their metabolites [32]. Of note, the proportion of correlations between the neurotransmitter and its metabolite in single brain region (Supplementary File, Figure S2) was similar to that previously reported in mice [25] or rats [32,33]. It reached approximately 50% of brain regions with significant correlations. It was lower for the link between VMA and NA acknowledging that VMA is one of the four metabolites of NA, and perhaps not the main one [34]. While the proportion of the metabolic link for DA and 5-HT systems was similar (50%) in both genotypes, the pattern slightly changed in CD mice. For NA and VMA, the proportion increased, suggesting higher coherence of the metabolic activity for NA terminals in CD mice.

The correlations of the DOPAC/DA ratio were reduced across the brain, but their pattern in balance with the 5-HIAA/5-HT ratio evolved from widespread in WT to nucleus accumbens/striatum distribution in CD mice. An opposite organization was seen for the interaction between DOPAC/DA and VMA/NA, where correlative links centered in the hippocampus and thalamus in WT became more widespread in CD mice. Overall, these changes of correlation patterns for the neuromodulatory systems in CD mice suggest a distinct involvement of neurobiological networks in behavioral strategies compared to WT, potentially associated with altered behavioral traits [23].

The behavioral deficits displayed by CD mice involve a large variety of domains [7,10], and can be observed since early life phases [10] throughout adulthood [7,9,12,13,14,15]. It is not our intent to discuss all of them in view of our data. The possible interference of the monoaminergic profile we report could speculatively concern alteration of exploratory behaviors, altered social interaction and communication, and sensorial sensitivity. Regarding the former, the decrease in accumbal DA content, often associated with reduced motivation, reward, and/or lower motor responses [18,21] is in line with the reduced locomotor activity described in the open field test in adult CD mice [14]. It is noteworthy that DOPAC/DA in prelimbic cortex, known to be increased after acute electric shock or open field in mice [35,36], is decreased in CD mice, presumably revealing lower DA cortical tone. This deficit could favor subcortical DA tone, but this one is limited by lower availability of accumbal DA. We recently reported that exploratory behavior in mice was associated with an increase in DA correlations organized around the prelimbic and cingulate cortex, striatum and nucleus accumbens, the ventral tegmental area, and the cerebellum over 28 sampled regions [27]. Therefore, lower DA tissue content in the nucleus accumbens and DOPAC/DA in the prefrontal cortex suggest altered capability of mesocorticolimbic neurons to respond to cues in novel environmental demands, which could include social cues as suggested previously [11]. The deficits in DA could also sustain impaired fear responses in a conditioned fear task [11].

Regarding the hypersocial behavior, the decrease in hypothalamic 5-HT metabolic activity, possibly in association with the lower accumbal DA levels evoked above, could participate in the heightened social, affiliative behavior [10]. Indeed, the hypothalamus, particularly in its ventral parts, is involved with numerous brain regions in social behavior, interaction with congeners, and aggressiveness [37,38,39,40]. It is not intuitive as low 5-HT level is often associated with aggressiveness [41], but it is remarkable that the decrease in 5-HT occurred only in CD mice. Intriguingly, increased DOPAC/DA were described in the cortex, striatum, and hippocampus [42,43] of the *Fmr1*-KO mouse model of autism spectrum disorder, characterized by social deficits opposite to the hypersociability of CD mice [44]. Thus, the decrease in 5-HT biochemical activity in the hypothalamus can offer other perspectives on the link between 5-HT, social behavior, and hypothalamus. The nucleus accumbens also play a role in modulating affiliative behaviors with a possible involvement of 5-HT [45]. The higher proportion of correlations of the DA and 5-HT ratios in the nucleus accumbens and the striatum of CD mice might be a tribute to such a hypothesis.

The last comment concerns the higher sensorial sensitivity of CD mice marked notably by higher auditory acuity [46]. The neurochemical study indicates that NA activity is not deficient in CD mice, NA neurons being strongly involved in sensory responses and arousal [19,47,48]. The anticipated lower DA responses to novel environment would enhance the brain NA responses to sensory stimuli, the NA systems participating also in exploratory behavior with distinct fingerprint compared to DA [27]. Concerning hyperacusis, we selected the auditory cortex, but we report no quantitative changes of monoaminergic systems, and saw only changes in correlative profiles between CD and WT.

The neurochemical dataset could have beneficiated with a higher number of animals, which corresponded here to the minimum number of observations we previously fixed arbitrarily [23]. A larger sample size would have been necessary to correlate monoamine levels with the behavioral alterations of CD mice, an issue that was beyond the scope of our study, lying in the evaluation of baseline monoamine function in behaviorally naïve mice. An additional limitation of our study was the lack of experiments in age-matched male animals which were not available. The behavioral alterations are similar in CD males and females at adulthood [7,14], but we cannot fully exclude sex-dependent differences in brain monoaminergic alterations. Nonetheless, our findings provide the first proof of evidence of the presence of specific alterations of monoaminergic function in the CD mouse model of WBS. These data complete with neurochemical results the anatomical and electrophysiological data showing altered development of neurons in several brain regions [7,10], or synaptic plasticity in hippocampus [12]. These changes suggest a modification of brain functioning in CD mice at rest and are in line with the abnormal coordination of brain regions that has been hypothesized in WBS.

## 5. Conclusions

Our findings show that female CD mice display specific alterations of monoamine content as well as a modification of the patterns of correlations among the different brain regions. The DA defects seem to be pivotal for the remodeling of the connectivity of monoaminergic systems. This study constitutes the first evidence of a modification of monoaminergic systems in the CD model of WBS that could play a role in the numerous behavioral disturbances reported in the CD model. One may further these data by addressing the neurochemistry of monoaminergic systems during neurodevelopment because behavioral alterations occur early in both males and females. It would be required in view of considering monoaminergic-based medication in WBS to mitigate monoaminergic imbalance we report, possibly largely before adulthood.

## Data Availability

The dataset is available upon request.

## Supplementary Materials

The following supporting information can be downloaded at https://www.mdpi.com/article/10.3390/biom15101382/s1, One supplementary file contains two figures corresponding to additional data that are not shown in the main text. The Figure S1 reports the concentration of HVA, L-DOPA and 5-HTP in the studied brain regions of WT and CD mice. The Figure S2 reports the correlative link between the neurotransmitter and its metabolite in single brain regions of WT and CD mice.

## Abbreviations

The following abbreviations are used in this manuscript:

5-HIAA	5-hydroxyindoleacetic acid
5-HT	5-hydroxytryptamine
5-HTP	5-hydroxytryptophan
A	Amygdala
AuC	Auditory cortex
CD	Complete deletion
CNS	Central nervous system
DA	Dopamine
dH	Dorsal hippocampus
DOPAC	3,4-Dihydroxyphenylacetic acid
HPLC	High pressure liquid chromatography
HVA	Homovanillic acid
Hy	Hypothalamus
L-DOPA	L-3,4-dihydroxyphenylalanine
NA	Noradrenaline
NAc	Nucleus accumbens
OF	Orbitofrontal Cortex
PL	Prelimbic Cortex
STR	Striatum
T	Thalamus
vH	Ventral hippocampus
VMA	Vanillylmandelic acid
WBS	Williams–Beuren Syndrome
WT	Wild type
