# Supplementary material for: Brain Monoamine Deficits in the CD Mouse Model of Williams–Beuren Syndrome
Source: Biomolecules. 2025 Sep 28;15(10):1382. doi: 10.3390/biom15101382 (PMC12562208; doi:10.3390/biom15101382)
Supplement: Supplementary file 1 [file biomolecules-15-01382-s001.zip › biomolecules-3865824-supplementary.pdf]

Supplementary File.

Brain Monoamine Deficits in the CD Mouse Model of Williams-Beuren Syndrome

Chloé Aman [1], Hélène Gréa [1], Alicia Rousseau [1], Anne-Emilie Allain [1], Susanna Pietropaolo [1], Philippe De Deurwaerdère [1*], Valérie Lemaire [1].

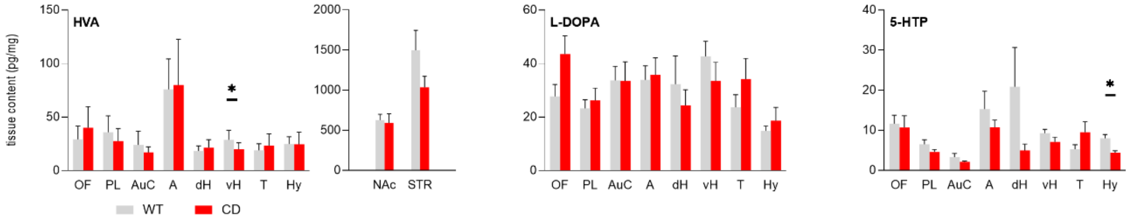

Supplementary File Figure S1. Monoamine precursor and metabolite content in wild-type and CD mice in sampled brain regions. Bar charts illustrate the tissue content of DA metabolite (left : HVA), and monoamine precursor (middle, right : L-DOPA, 5-HTP) across the brain (OF: orbitofrontal cortex, PL: prelimbic cortex, AuC: auditory cortex, A: amygdala, dH: dorsal hippocampus, vH: ventral hippocampus, NAc: nucleus accumbens, STR: striatum, T: thalamus, Hy: hypothalamus). WT (light gray) and CD (red). L-DOPA and 5-HTP were not measured in STR and NAc. Data are expressed as mean ± SEM. **p <0.05, Student t-test* . WT (n = 9), CD (n=7).

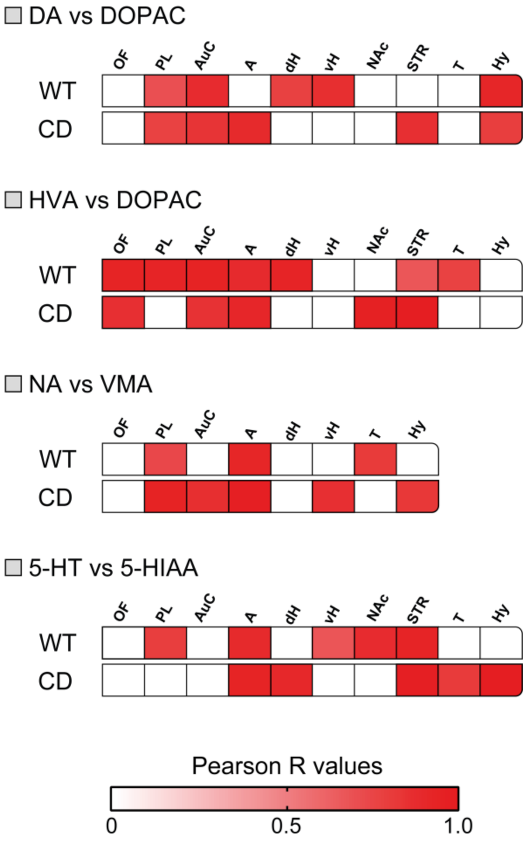

Supplementary file figure 2: Interdependency between monoamines and their relative metabolites within the brain regions in WT and CD mice. Correlation between monoamine and their metabolites for WT and CD mice for each brain regions (OF: orbitofrontal cortex, PL: prelimbic cortex, AuC: auditory cortex, A: amygdala, dH: dorsal hippocampus, vH: ventral hippocampus, NAc: nucleus accumbens, STR: striatum, T: thalamus, Hy: hypothalamus). Each red square corresponds to a significative and positive correlation ($p < 0.05$, r Pearson's coefficient. The color gradient corresponds to the strength of the correlation (R Values).
